# Whole blood human transcriptome and virome analysis of ME/CFS patients experiencing post-exertional malaise following cardiopulmonary exercise testing

**DOI:** 10.1371/journal.pone.0212193

**Published:** 2019-03-21

**Authors:** Jerome Bouquet, Tony Li, Jennifer L. Gardy, Xiaoying Kang, Staci Stevens, Jared Stevens, Mark VanNess, Christopher Snell, James Potts, Ruth R. Miller, Muhammad Morshed, Mark McCabe, Shoshana Parker, Miguel Uyaguari, Patrick Tang, Theodore Steiner, Wee-Shian Chan, Astrid-Marie De Souza, Andre Mattman, David M. Patrick, Charles Y. Chiu

**Affiliations:** 1 Department of Laboratory Medicine, University of California San Francisco, San Francisco, California, United States of America; 2 Communicable Disease Prevention and Control Services, Vancouver, Canada; 3 School of Population and Public Health, University of British Columbia, Vancouver, Canada; 4 Workwell Foundation, Ripon, California, United States of America; 5 Department of Pediatrics, Division of Cardiology, University of British Columbia, Vancouver, Canada; 6 British Columbia Centre for Disease Control Public Health Laboratory, Vancouver, Canada; 7 Department of Pathology and Laboratory Medicine, University of British Columbia, Vancouver, Canada; 8 Centre for Health Evaluation Outcome Sciences, Vancouver, Canada; 9 Department of Pathology, Sidra Medical and Research Center, Doha, Qatar; 10 Department of Medicine, Division of Infectious Diseases, University of British Columbia, Vancouver, Canada; 11 Division of Cardiology, British Columbia’s Children’s Hospital, Vancouver, Canada; 12 Adult Metabolic Disease Clinic, Vancouver General Hospital, Vancouver, Canada; 13 Department of Medicine, Division of Infectious Diseases, University of California San Francisco, San Francisco, California, United States of America; Centers for Disease Control and Prevention, UNITED STATES

## Abstract

Myalgic encephalomyelitis / chronic fatigue syndrome (ME/CFS) is a syndrome of unknown etiology characterized by profound fatigue exacerbated by physical activity, also known as post-exertional malaise (PEM). Previously, we did not detect evidence of immune dysregulation or virus reactivation outside of PEM periods. Here we sought to determine whether cardiopulmonary exercise stress testing of ME/CFS patients could trigger such changes. ME/CFS patients (n = 14) and matched sedentary controls (n = 11) were subjected to cardiopulmonary exercise on 2 consecutive days and followed up to 7 days post-exercise, and longitudinal whole blood samples analyzed by RNA-seq. Although ME/CFS patients showed significant worsening of symptoms following exercise versus controls, with 8 of 14 ME/CFS patients showing reduced oxygen consumption (${\dot{\text{V}}\text{O}}_{2}$) on day 2, transcriptome analysis yielded only 6 differentially expressed gene (DEG) candidates when comparing ME/CFS patients to controls across all time points. None of the DEGs were related to immune signaling, and no DEGs were found in ME/CFS patients before and after exercise. Virome composition (P = 0.746 by chi-square test) and number of viral reads (P = 0.098 by paired t-test) were not significantly associated with PEM. These observations do not support transcriptionally-mediated immune cell dysregulation or viral reactivation in ME/CFS patients during symptomatic PEM episodes.

## Introduction

Myalgic encephalomyelitis/ chronic fatigue syndrome (ME/CFS) is characterized by long-term, debilitating fatigue that is characteristically exacerbated by physical and mental exertion [1,2]. Patients also typically experience impaired sleep, cognitive complaints, myalgia, arthralgia, headache, and other symptoms. Patients with ME/CFS have more unmet medical and home care needs and greater functional disability overall than those with other chronic disorders [3,4]. Post-exertional malaise (PEM) is a key symptom of ME/CFS and is described as a cluster of symptoms following mental or physical exertion, often involving a loss of physical or mental stamina, rapid muscle or cognitive fatigability, and sometimes lasting 24 hours or more [2]. These symptoms form the basis for a recent redefinition of the condition [1].

No specific cause for ME/CFS has been identified. Diagnosis of the syndrome based on symptoms and exclusion of other diseases in the absence of established biomarkers contributes to inconsistent clinical case definitions [5]. Working etiological hypotheses have included roles for viruses, bacteria, environmental triggers, immune dysregulation, mitochondrial dysfunction and disorders of oxidative metabolism [2,6,7]. Several lines of evidence point toward a role for disordered immunity or inflammation. These include disordered cytokine expression in serum [8] and cerebrospinal fluid [9], NK cell dysfunction [10] and promising response to early trials of B cell depletion [11]. Separately, these results suggest that irregularities in circulating immune cell subsets, and their gene expression, drive chonic autoimmune activation. Dysregulation of the immune response can also lead to increases in viral titers [12,13]. ME/CFS has been associated with reactivation of various viral infection [14]. Taken together these studies do not validate each other, but rather describe heterogeneous putative disease mechanisms [15].

RNA-seq can be leveraged to identify and count at once all transcripts and RNA genomes within a sample. This method has been used to discover host biomarkers and study molecular pathways involved in disease response [16], as well as for the identification of known and unsuspected infectious agents [17]. Using RNA-seq, we previously published data comparing gene expression profiles of patients with ME/CFS against a matched control group [18]. No differentially expressed genes and no differences in the blood virome were found to be associated with ME/CFS in the resting state. One 2014 review concluded that there may be altered immune responses to exercise in ME/CFS [19]. We launched the current study to provide an experimental approach to search for differential gene expression and changes in viral abundance induced by cardiopulmonary exercise testing (CPET) in association with the cardinal symptom of PEM. This approach also provides the benefit of allowing objective subtyping of patients according to exercise response phenotype [20].

## Methods

### Ethics, consent, and permissions

Our study protocol was approved by the Institutional Review Board at the University of British Columbia (Certificate # H14-01149). Potential subjects were recruited through postings to relevant newsletters and websites and information made available by participating physicians. Informed consent was obtained from all participants and/or their legal surrogates. Written informed consent included a full explanation of how the CPET regimen had been designed to provide an objectively measurable physiological stimulus to induce fatigue. Healthy people were matched with ME/CFS subjects by 5-year age stratum, ethnicity and gender. Patients were excluded from any group if they did not provide consent, were <18 years of age, were unable to understand English, or if they had another medical condition that accounted for their main symptoms. Any specific diagnosis that would exclude a person from a diagnosis of ME/CFS also excluded a healthy person from being recruited as a control. Because our sample size in this small pilot study was too small to address gender related differences, we recruited female cases and controls only.

### Study cohort

We recruited female patients with ME/CFS (Canadian 2003 criteria) [21] and age- and gender-matched sedentary controls. Controls met the American College of Sport Medicine criteria for sedentary lifestyle, not participating in a regular exercise program nor accumulating 30 minutes or more of moderate physical activity on most days of the week [22]. After informed consent, subjects completed study questionnaires including demographics, history, SF 36 [23], Fatigue Severity Scale [24], a Functional Capacity Scale used widely by ME/CFS practitioners [25], Karnofsky [26], CESD [27], State Trait Anxiety Inventory (STAI) [28], Physical Activity Scale for the Elderly (PASE) [29] and the Pittsburgh Sleep Quality Index [30] and underwent a screening physical examination and screening laboratory tests. Important elements of the history included the acuity of onset of symptoms, duration of diagnosis of ME/CFS, history of a classical infectious illness at onset, and gender. Screening laboratories included tests for complete blood count (CBC), C-reactive protein (CRP); calcium, random glucose; lactate; magnesium; phosphate; sodium; potassium; chloride; total carbon dioxide level (CO_2_); urea; creatinine; total bilirubin; uric acid; alanine transaminase (ALT); aspartate transaminase (AST); creatine kinase (CK); gamma-glutamyl transferase (GGT); albumin; rheumatoid factor; thyroid stimulating hormone (TSH); ferritin; anti-nuclear antibody (ANA), total protein with protein electrophoresis; human immunodeficiency virus (HIV); hepatitis B virus (HBV); hepatitis C virus (HCV); syphilis; and two-tiered Lyme serology. A complete physical examination was conducted with a strong focus on musculoskeletal and neurological findings. All baseline data were reviewed to assure that case definitions were met.

### Baseline accelerometry

Before enrollment, cases and controls wore a wGT3X-BT accelerometer (ActigraphCorp, Pensacola FL) on an elasticized belt around their waist for a period of five days, including one weekend. The device records human activity in three axes. Participants were asked to remove the device in order to shower or bathe but to wear it at all other times, including sleep periods. Parameters provided by the accelerometer report include: mean percent of time it was worn, total steps, percent of time spent in sedentary, light, moderate, vigorous and very vigorous activity, time in bed, time sleeping and number of awakenings.

### Cardiopulmonary exercise testing

The protocol called for each subject to undergo two maximal cardiopulmonary exercise tests (CPET) approximately 24h apart. Subjects were fitted with electrocardiogram (ECG) electrodes for monitoring of heart rhythm, a mouthpiece and headgear for collection of expired air, and a pulse oximeter for monitoring arterial oxygen saturation. Subjects were allowed to pedal for a short period (less than one minute) prior to an incremental exercise test performed on an electronically braked bicycle ergometer (Lode Excalibur Sport, Lode BV, Groningen, the Netherlands). Workload was increased 15 watts per minute until volitional fatigue. ECG was monitored continuously for signs of cardiac arrhythmia or ischemia, and pulse oximetry was monitored to ensure safe levels of arterial oxygenation. Blood pressure was regularly monitored for patient safety. Expired respiratory gases were collected using a metabolic cart (Moxus System, AEI Technologies, Pittsburgh, PA) and minute ventilation ($\dot{\text{V}}\text{E}$, L/min), oxygen consumption (${\dot{\text{V}}\text{O}}_{2}$, L/min or mL/min/kg), carbon dioxide production (${\dot{\text{V}}\text{CO}}_{2}$, L/min), the respiratory exchange ratio (RER), and the ventilatory equivalents for oxygen ($\dot{\text{V}}\text{E}$/(${\dot{\text{V}}\text{O}}_{2}$) and carbon dioxide ($\dot{\text{V}}\text{E}$/(${\dot{\text{V}}\text{CO}}_{2}$) were calculated. The ventilatory threshold was calculated using the V-slope method [31]. Subjects were encouraged to pedal as long as possible and testing was terminated when criteria for maximal effort were met (according to American College of Sports Medicine Guidelines or upon participant self-reported exhaustion). Subjects then remained seated on the ergometer through recovery for 10 minutes. Questionnaires on fatigue and other experienced symptoms were administered before each exercise test, within 15 minutes of completion of each test and at days 3 and 7 of follow-up.

### Sample collection, preparation and sequencing

Study blood for total RNA was collected at day 1 and day 2 within 2 hours prior to each exercise test session, and during a home visit on day 3 and day 7. Each subject thus contributed 4 whole blood samples for RNA-seq analysis. Whole blood (2.5 mL) from cases and controls was drawn into a PAXgene Blood RNA Tube (Qiagen, Valencia, CA) to stabilize RNA prior to extraction and stored at -20°C. Total RNA was extracted at University of British Columbia using the PAXgene Blood RNA Kit (Qiagen, Valencia, CA) and all samples were lyophilized in RNAstable reagent (Biomatrica, San Diego, CA) for shipment at room temperature to University of California, San Francisco (UCSF) for further processing and long-term storage. The Ovation Human blood RNA-seq kit (Nugen, San Carlos, CA) was used to generate strand-specific RNA-seq libraries depleted for reads derived from rRNA (12S, 16S, 18S and 28S genes) and globin (HBA1, HBA2, HBB and HBD genes) according to the manufacturer’s protocol. The Ovation RNA-seq kit employs depletion of human RNA and globin transcripts, which can constitute up to 76% of mRNA in whole blood [32]; in our experience, up to a 4-fold increase in sensitivity for informative transcripts is observed using this kit. Briefly, 100ng of RNA extract, as measured by Qubit RNA high sensitivity kit (Thermo Fisher, South San Francisco), were treated with DNase according to the manufacturer’s instructions, and cDNA was prepared from extracted total RNA by reverse transcription using a mixture of random and poly(T) primers. Successive steps of end-repair, adaptor ligation, strand selection via nucleotide analog-targeted degradation, insert-dependent adaptor cleavage for targeted depletion of rRNA and globin reads, PCR amplification, and bead-based purification were then used to construct cDNA libraries for RNA-seq analysis. The 100 resulting libraries were assessed for quality using the Agilent Bioanalyzer DNA High Sensitivity Kit (Agilent, Santa Clara, CA) and sequenced as 100 base pair (bp) paired-end runs across 8 lanes on a HiSeq 2500 instrument (Illumina, San Diego, CA). No ERCC (External RNA Control Consortium) spike-ins were added to the libraries; however, no batch effect was apparent by principal component analysis (PCA).

#### Transcriptome analysis

Gene expression analysis was conducted using Partek Flow software (version 5.0). Paired-end reads were mapped to the human genome (hg38), annotated to exons, and normalized to FPKM (fragments per kilobase of exon per million fragments mapped) values for all 25,278 human RNA reference sequences in the National Center for Biotechnology Information (NCBI) RefSeq database (August 2016 build) using STAR v2.4.1 [33] and Cufflinks v2.2.1 [34]. Differential expression of genes was calculated using a multimodel approach (normal, lognormal, lognormal with shrinkage, negative binomial and Poisson distributions) in which the best model fit for each gene is chosen based on the Akaike information criterion corrected for small sample sizes [35]. Genes were considered to be differentially expressed when their fold change was > ± 1.5, *p*-value < 0.05, and adjusted *p*-value (or false discovery rate, FDR) < 0.1%.

### Viral metagenomic analysis

Sequencing data from whole transcriptome libraries were analyzed for the presence of RNA sequences corresponding to known human viral pathogens using the sequence-based ultra-rapid pathogen identification (SURPI) computational pipeline [36]. After computationally subtracting human reads, remaining reads were aligned against all microbial sequences in the National Center for Biotechnology Information (NCBI) GenBank reference database. The SNAP aligner [37] was used at moderate stringency (edit distance = 12) to align reads to the NCBI nucleotide nt database, allowing for detection of reads with ≥90% nucleotide identity to known viruses, while RAPSearch [38] was used to detect divergent reads from potential novel viruses by translated nucleotide alignment to the NCBI protein nr database. A rapid taxonomic classification algorithm based on the lowest common ancestor was incorporated into SURPI, as previously described [39], and used to assign viral reads to the species, genus, or family level.

### Clinical and epidemiological analysis

All clinical, laboratory and exercise data were anonymized to study number and stored securely in a common linked database at the University of British Columbia. Descriptive statistical tests were performed in R. To avoid the assumption that the data fit a fixed probability distribution, univariate analyses comparing groups were performed using non-parametric methods; a Fisher’s Exact Test was used for categorical variables and a Mann-Whitley rank sum test for continuous variables.

ME/CFS patients were classified according to whether or not they had a significant decline in performance on the second exercise test defined as ≥ 7% decrease in ${\dot{\text{V}}\text{O}}_{2}$ at either the peak or at the ventilatory threshold. CPET test results were provided to a group of investigators (SS, JS, MVN, CS) who were blinded as to case and control status in order to allow expert identification of a test-retest effect on repeat CPET.

## Results

### Study cohort

The CPET study screened 136 potential participants and enrolled a total of 25 subjects (14 cases, 11 controls). The potential participants who were not enrolled included those who were lost to follow-up (n = 2), withdrew from the study prior to exercise testing (n = 2), were unable to participate due to the lack of eligible age-matched controls or cases (n = 26), declined to participate (n = 40, many who wished to avoid triggering symptoms of exercise-induced PEM), or were ineligible according to Canadian Consensus Criteria because of the presence of an underlying illness precluding a diagnosis of ME/CFS (n = 41). Seven out of the fifteen ME/CFS subjects had also participated in a previous published study examining gene expression profiles in comparison to a matched control group [18]; however, the CPET study was a separate study with its own enrollment cohort.

Characteristics of our 14 cases and 11 matched controls are shown in Table 1. Patients were significantly matched to controls for age, gender and body mass index (*p* > 0.62). Cases had more defining symptoms and scored lower on disability scales than did controls (*p* < 0.01). Nine cases described a history of an infectious prodrome at disease onset, manifesting as an acute “flu-like illness” or gastroenteritis, while five described a sudden onset of ME/CFS. Baseline accelerometry revealed that sedentary controls trended toward logging more steps over a five-day period than did ME/CFS subject (Table 1), albeit not reaching statistical significance. However, the distribution of time spent on different levels of activity (sedentary, light, moderate and vigorous exercise) was similar between control and ME/CFS patients. While controls trended toward logging even more awakenings per night than cases, they slept more over a 24-hour cycle; these differences did not reach statistical significance.

**Table 1:** Characteristics of the study population.

Variable	ME/CFS (n = 14)	Control (n = 11)	*P* Value
***Age in years*, *median (IQR)***	49 (38, 58.3)	50 (45.5, 58.5)	0.6216
***BMI*, *median (IQR)***	26.63 (21.4, 34.9)	26.45 (24.1, 35.2)	0.7675
***Core Symptoms*, *n (%)***			
Fatigue	14 (100)	1 (9)	**< .00001**
Pain	14 (100)	3 (27)	**< .001**
Post Exertional Fatigue	14 (100)	1 (9)	**< .00001**
Karnofsky Score Last 7 Days	60 (56.25, 68.75)	100 (90, 100)	**< .0001**
Functional Capacity Last 7 Days	4 (3, 5.75)	8 (7.75, 8)	**< .0001**
***Illness Onset Pattern n (%)***			
Gradual	9 (64)	NA	NA
Sudden	5 (36)	NA	NA
History of Infection at Onset	9 (64)	NA	NA
Illness duration yrs, median (IQR)	5.75 (5, 7.5)	NA	NA
***Baseline 5 Day Accelerometry*, *Median(IQR)***			
Wear Time (% of time)	90.5 (89, 94)	95 (93, 96)	0.1958
Steps in 5 Days	28447(15722,33186)	32995 (20940,50688)	0.2671
Activity Distribution (% of time)			
*Sedentary*	78.4 (71,81.5)	74.5 (71.1,77.2)	0.3111
*Light*	21.05 (18,26)	22.8 (20.5,24.6)	0.5719
*Moderate*	1.1 (0.3,2.1)	1.3 (0.8,4.2)	0.2612
*Vigorous*	0.0 (0, 0)	0.0 (0, 0)	0.8084
Sleeping (Hours of Sleep Per 24 Hours)	6.7 (5.9, 7.9)	7.3 (6.2, 7.9)	0.4593
Awakenings (Per 24 Hours)	5.8 (4.6,6.9)	8.2 (5.3, 10)	0.0708

Values are presented as median (IQR). *P* values were calculated with Wilcoxon rank-sum test. Statistically significant values (*p* < 0.05) were denoted in bold.

### Cardiopulmonary exercise results

On day 2 of exercise testing, controls exercised for longer periods of time, achieved higher peak power output, and achieved higher peak ${\dot{\text{V}}\text{O}}_{2}$ than ME/CFS participants (Table 2). Heart rate and blood pressure responses were similar between the groups, while ME/CFS patients self-reported higher scores for perceived exertion. In comparing the second to the first day of exercise testing, those with ME/CFS were able to exercise for significantly less time while controls actually increased their exercise time on the second day (p = 0.0299).

**Table 2:** Cardiopulmonary exercise test parameters in CFS patients compared to controls on 2 consecutive days.

Measure (unit)	Day 1			Day 2^†^			Day 2 vs. Day 1		
	Case (n = 14)	Control (n = 11)	*P*	Case (n = 12)	Control (n = 10)	*P*	Case (n = 12)	Control (n = 10)	*P*
**Exercise Time (s)**	467.0 (447.2, 579.8)	556.0 (533.0, 618.5)	0.1072	444.5 (418.2, 520.5)	573.5 (535.8, 648.5)	**0.0161**	- 23.0 (-36.5, 2.0)	13.0 (-4.5, 28.5)	**0.0479**
**Peak Power Output (watts)**	120.0 (105.0, 146.2)	135.0 (135.0, 165.0)	0.0539	105.0 (105.0, 135.0)	142.5 (135.0, 165.0)	**0.0188**	0.0 (-15.0, 0.0)	0.0 (0.0, 0.0)	0.1151
**Metabolic Equivalent Units**	6.6 (5.9, 8.1)	7.3 (5.5, 8.1)	0.6443	6.1 (5.2, 7.3)	7.1 (5.9, 7.7)	0.2622	-0.5 (-1.1, -0.1)	-0.1 (-0.4, 0.0)	0.1964
**Peak Heart Rate (bpm)**	160.0 (143.5, 182.0)	157.0 (153.5, 164.0)	0.6606	153.5 (141.2, 170.0)	160.0 (154.0, 162.0)	0.4674	-3.0 (-7.0, 1.5)	1.0 (-2.8, 3.0)	0.2595
**Peak Systolic Blood Pressure (mmHg)**	178.0 (160.0, 196.0)	184.0 (171.0, 201.0)	0.3379	177.0 (154.2, 193.0)	185.0 (175.0, 191.5)	0.3064	-4.0 (-6.0, 12)	6.0 (-4.0, 13.0)	0.8321
**Peak Diastolic Blood Pressure (mmHg)**	88.0 (80.0, 94.0)	86.0 (82.5, 91.5)	0.8274	85.0 (79.0, 90.5)	87.0 (84.0, 91.5)	0.5491	-2.0 (-2.0, 0.0)	2.0 (-2.0, 4.0)	0.3969
**Lowest Oxygen Saturation (%)**	96.0 (95.0, 98.0)	96.0 (93.5, 96.5)	0.3748	97.0 (95.5, 98.0)	96 (94.3, 97.8)	0.5212	0.0 (-1.5, 1.5)	1.0 (-0.8, 2.5)	0.6695
Rate of Perceived Exertion*	19.0 (19.0, 20.0)	17.0 (14.5, 18.0)	**0.0145**	19.0 (17.8, 20.0)	17.0 (15.0, 19.0)	0.0973	0.0 (-0.3, 0.3)	0.5 (-1.5, 1.0)	0.6829
**Minute Ventilation (L/min)**	64.1 (49.7, 78.9)	79.2 (63.4, 83.5)	0.1056	62.4 (54.9, 70.6)	71.8 (64.1, 86.4)	0.2030	-0.9 (-6.1, 6.7)	-2.0 (-5.7, 2.6)	0.4287
**Absolute Peak ${\dot{\text{V}}\text{O}}_{2}$ (mL/min)**	1557 (1474, 1795)	1792 (1698, 1914)	0.2066	1460 (1344, 1566)	1679 (1646, 1846)	**0.0426**	-108.5 (-243.5, -18.5)	-30.5, (-73.0, 6.8)	0.1562
**Relative Peak ${\dot{\text{V}}\text{O}}_{2}$ (mL/min/kg)**	23.2 (20.6, 28.3)	25.6 (19.2, 28.2)	0.8393	22.0 (18.1, 25.5)	24.6 (20.7, 26.9)	0.2543	-1.6 (-3.8, -0.3)	-0.4 (-1.5, 0.1)	0.1983
**Relative ${\dot{\text{V}}\text{O}}_{2}$ at the Ventilatory Threshold** **(mL/min/kg)**	13.5 (7.3, 19.8)	12.0 (9.5, 15.0)	0.4761	14.5 (5.8, 20.3)	11.5 (8.3, 14.8)	0.6277	4.5 (-1.0, 6.0)	1.5 (-1.0, 5.3)	0.6425
**Absolute Peak ${\dot{\text{V}}\text{CO}}_{2}$ (mL/min)**	1814 (1684, 2241)	2171 (1880, 2270)	0.2767	1688 (1566, 1832)	2146 (1910, 2332)	**0.0249**	-164.5 (-292.8, -17.3)	9.0 (-38.3, 46.3)	0.1402
**Respiratory Exchange Ratio**	1.16 (1.04, 1.26)	1.15 (1.12, 1.21)	1	1.16 (1.11, 1.26)	1.19 (1.14, 1.27)	0.6918	0.02 (-0.01, 0.04)	0.04 (-0.03, 0.08)	0.7414
**Subjects with ≥7% decline in peak ${\dot{\text{V}}\text{O}}_{2}$ or ${\dot{\text{V}}\text{O}}_{2}$ at the ventilatory threshold day 2 vs day 1**							8	4	
Test/Retest Effect**							7	4	

Values are presented as median (IQR). *P* values were calculated with Wilcoxon rank-sum test. Statistically significant values (*p* < 0.05) were denoted in bold.

^†^: Three subjects did not complete the second day of testing, two (1 case and 1 control) for safety reasons (high blood pressure response) and another (case) due to technical problems.

* Perceived effort measured by modified Borg scale

** Blinded expert reading of full spectrum of repeat CPET parameters indicated a test/retest effect.

Two subjects (one case and one control) successfully completed only one exercise test because systolic blood pressure was found to be high after testing. These subjects were retained for the gene expression analysis because follow-up confirmed the development of PEM for the patient with ME/CFS.

We identified eight ME/CFS subjects with a significant decline in performance from day 1 to day 2 based on a decrease in ${\dot{\text{V}}\text{O}}_{2}$ at either the peak or at the ventilatory threshold. Seven of these eight were also assigned a test-retest effect based on blinded expert review of full test data.

**Fig 1:**
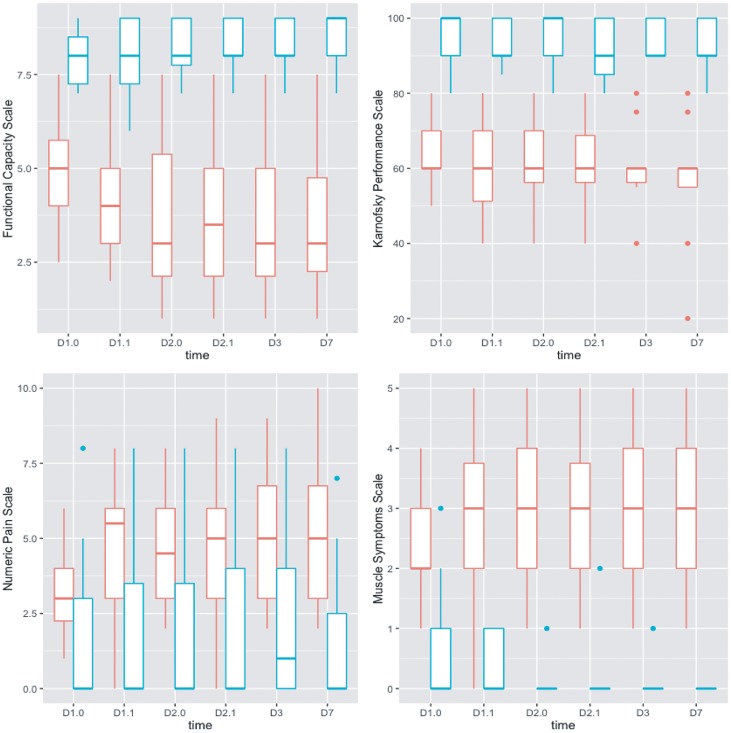
Change of numeric scales for functional scores, Karnofsky performance, pain and muscle symptoms over time. CFS patients = red, matched controls = green. D1.0 and D2.0 indicate pre-exercise assessments on day 1 and day 2, and D1.1 and D2.1 indicate post-exercise assessments on day 1 and day 2. D3 and D7 are no-exercise follow-up results.

Participants with ME/CFS but not controls reported a worsening of symptoms following exercise that became maximal by day 3 or day 7 on most scales (Fig 1). This was most clear with a decrease in the Functional Capacity Scale and by increases in numeric pain and muscle symptom scores.

### Differential gene expression by transcriptome analysis

**Fig 2:**
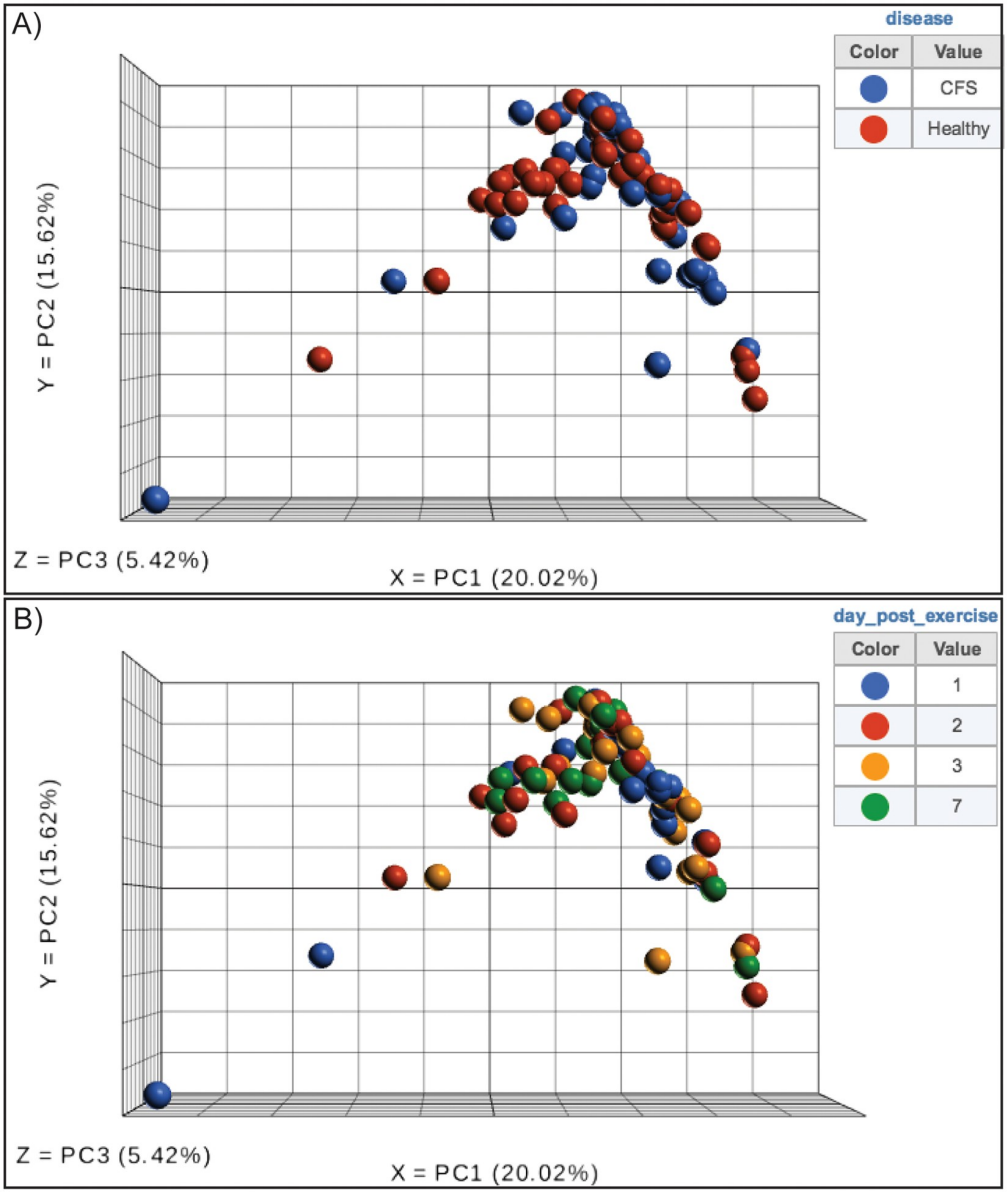
Principal component analysis of the global gene expression shows no sampling bias between CFS patients and controls (A), nor between time points (B).

An average of 15.4 million reads per sample were generated (IQR: 13.4–19.4 million reads per sample), with an average of 78% uniquely mapping reads and average transcriptome coverage of 68%. Two samples from a single subject (day 3 and 7 samples) had unusually low number of expressed genes detected (< 40%) and thus were removed from the analysis (**Fig A in** S1 Appendix). Principal component analysis of the whole transcriptome showed no clustering of samples on the basis of disease status or study time point (Fig 2). No clear clustering based on library preparation batch was observed by PCA (**Fig B in** S1 Appendix). Additionally, samples had been blinded and randomized during processing of the sample.

No DEGs were detected when comparing pre- to post-exercise samples in ME/CFS as compared to controls, both overall (days 1, 2 versus day 3, 7) or at any single time point (Table 3). Six DEGs were detected comparing ME/CFS to controls at all time points combined, and 1 DEG was detected at day 7 alone (Table 3). One to three DEGs were detected when comparing the low ${\dot{\text{V}}\text{O}}_{2}$ subset of ME/CFS patients against subjects with regular re-test ${\dot{\text{V}}\text{O}}_{2}$ at days 1, 3 and 7. In total, 19 unique DEGs were identified (Table 4, **Figs C-F in** S1 Appendix). Thirteen DEGs had average gene coverage lower than 1 normalized read/sample (CREB3L1, HFN4A-AS1, HOXA9, LINC01068, LINC01158, LOC100133050, LOC105372441, NRON, PMS2P2, PRR21, RNASE8, TMEM262, and USP50), and two DEGs were ribosomal genes targeted for depletion during RNA library preparation (RPL23A, RPS12). The other four DEGs correspond to small nucleolar RNAs SNORA27 and SNORA32, uncharacterized non-coding RNA LOC101928767, and MBIP (MAP3K12 binding inhibitory protein 1).

**Table 3:** Number of differentially expressed genes in ME/CFS patients compared to controls.

	**Timepoint**	**Total**	**Up-Regulated**	**Down-Regulated**
ME/CFS vs Controls	Day 1	0	0	0
	Day 2	0	0	0
	Day 3	0	0	0
	Day 7	0	0	1
	All days	6	4	2
Low ${\dot{\text{V}}\text{O}}_{2}$ ME/CFS subset vs Regular repeat exercise	Day 1	1	1	0
	Day 2	0	0	0
	Day 3	2	2	0
	Day 7	3	3	0
	All days	0	0	0
Test-retest effect ME/CFS subset vs Regular repeat exercise	Day 1	0	0	0
	Day 2	2	1	1
	Day 3	3	3	0
	Day 7	4	4	0
	All days	0	0	0
	**Disease**	**Total**	**Up-Regulated**	**Down-Regulated**
Day 1 vs Day 2	CFS	0	0	0
	HC	0	0	0
Day 1 vs Day 3	CFS	0	0	0
	HC	0	0	0
Day 1 vs Day 7	CFS	0	0	0
	HC	0	0	0

**Table 4:** List of differentially expressed genes in ME/CFS patients compared to controls.

Comparison	Gene ID	Gene name	Fold change	FDR	Gene count (avg.)
ME/CFS vs controls; all days	HOXA9	Homeobox A9	2.57	0.08	0.37
	LOC101928767	Uncharacterized	2.03	0.08	1.92
	NRON	Noncoding Repressor Of NFAT	1.53	0.08	0.36
	RPL23A	Ribosomal Protein L23a	-1.77	0.06	39.63
	RPS12	Ribosomal Protein S12	-1.56	0.02	646.28
	SNORA27	Small Nucleolar RNA, H/ACA Box 27	6.35	0.06	50.88
ME/CFS vs controls; Day 7	LINC01158	Long Intergenic Non-Protein Coding RNA 1158	1054	0.10	0.11
Lower ${\dot{\text{V}}\text{O}}_{2}$ vs Normal ${\dot{\text{V}}\text{O}}_{2}$; Day 1	LOC105372441	Uncharacterized	5745	3.72E-04	0.12
Lower ${\dot{\text{V}}\text{O}}_{2}$ vs Normal ${\dot{\text{V}}\text{O}}_{2}$; Day 2	LOC100133050	Glucuronidase Beta Pseudogene	6.42	1.19E-03	0.01
	PMS2P2	PMS1 Homolog 2, Mismatch Repair System Component Pseudogene 2	813	0.05	0.01
Lower ${\dot{\text{V}}\text{O}}_{2}$ vs Normal ${\dot{\text{V}}\text{O}}_{2}$; Day 7	PRR21	Proline Rich Protein 21	2072	1.98E-03	0.04
	TMEM262	Transmembrane Protein 262	11.07	1.98E-03	0.16
	USP50	Ubiquitin Specific Peptidase 50	1979	6.86E-03	0.04
Test-retest effect vs Regular exercise; Day 2	MBIP	MAP3K12 Binding Inhibitory Protein 1	-1.94	0.1	3.28
	SNORA32	Small Nucleolar RNA, H/ACA Box 32	726520	0.04	5.24
Test-retest effect vs Regular exercise; Day 3	LOC100133050	Glucuronidase Beta Pseudogene	4.7	0.04	0.01
	PMS2P2	PMS1 Homolog 2, Mismatch Repair System Component Pseudogene 2	947.93	4.81E-03	0.01
	RNASE8	Ribonuclease A Family Member 8	7956	0.04	0.12
Test-retest effect vs Regular exercise; Day 7	CREB3L1	CAMP Responsive Element Binding Protein 3 Like 1	5.82	0.04	0.04
	HNF4A-AS1	HNF4A Antisense RNA 1	2293	0.03	0.03
	LINC01068	Long Intergenic Non-Protein Coding RNA 1068	20.41	0.04	0.14
	TMEM262	Transmembrane Protein 262	14.06	1.19E-05	0.16

### Viral metagenomics

We detected sequences from a small number of viruses in the RNA-seq data, including enterovirus A, influenza A virus, anelloviruses/torque teno viruses (TTVs), and human herpesviruses (HHVs) (Table 5). Only one subject, matched control #01, showed an increase in the number of viral reads from day 1 to day 7, corresponding to anelloviruses. The overall virome composition in ME/CFS did not differ significantly from controls (P = 0.746 by chi-square test) and the number of viral reads did not significantly change in ME/CFS compared to controls (P = 0.098 by Welch’s t-test).

**Table 5:** Number of reads matching human viruses in positive samples by metagenomic RNA-seq.

Subject	Disease	Day	Enterovirus A	Human herpesvirus 1	Human herpesvirus 4	Human herpesvirus 6A	Human herpesvirus 6B	Human herpesvirus 7	Influenza A virus	Torque teno virus
Control #01	−	1	0	0	0	0	0	0	0	3
Control #01	−	2	0	2	0	0	0	0	0	6
Control #01	−	3	0	0	0	0	0	0	0	16
Control #01	−	7	0	0	0	0	0	0	0	18
Control #02	−	1	0	0	0	0	0	0	4	0
Control #06	−	3	0	4	0	0	0	0	0	0
Control #10	−	1	0	32	0	0	0	0	0	0
Control #10	−	2	0	1	0	0	0	0	0	0
Control #13	−	7	0	0	4	0	0	0	0	0
Control #14	−	7	17	0	0	0	0	6	0	0
Patient #02	ME/CFS	2	0	2	0	0	0	0	0	0
Patient #04	ME/CFS	3	0	2	0	0	0	0	0	0
Patient #05	ME/CFS	2	0	0	0	0	4	0	0	0
Patient #06	ME/CFS	2	0	16	0	0	0	0	0	0
Patient #08	ME/CFS	2	0	0	0	0	0	2	0	0
Patient #09	ME/CFS	2	0	0	0	0	0	0	1	0
Patient #09	ME/CFS	3	0	0	0	0	0	0	0	2
Patient #10	ME/CFS	2	0	0	0	2	0	0	0	0
Patient #10	ME/CFS	3	0	0	0	0	0	0	0	3
Patient #12	ME/CFS	2	0	0	0	0	0	0	0	1

## Discussion

This study builds on previous observations we have made in people with ME/CFS outside of PEM episodes [18]. While we documented profound clinical differences in health, disability and functional capacity in this population, we did not detect evidence of a transcriptionally-mediated immune mechanism driving the symptoms in ME/CFS. Here we sought to determine whether induced PEM in patients with ME/CFS following experimental CPET triggers differential immune responses and/or changes in the virome relative to controls and before and after exercise. Our rate of ME/CFS subjects enrolled among the screened population, 10.3% (14/136), is much higher than the self-reported prevalence of ME/CFS in Canada from national surveys of approximately 1.5% [4]. This is not unexpected as the screened population was enriched for subjects with symptoms of ME/CFS.

Overall, we did not find significant alterations in gene expression in patients with PEM relative to controls. Fifteen out of 19 DEGs identified in this study were likely false positives due to low gene coverage; the other four DEGs (MBIP, SNORA27, SNORA32 and LOC101928767) were of unclear function and/or showed borderline differences in gene expression. Patients with ME/CFS did also not show significant or relevant exercise-induced after exercise in ME/CFS using RT-PCR of targeted genes [40,41] and in discordant twins using microarrays [42], without identifying important DEGs. This study reinforces these observations by using a more comprehensive genome-wide RNA-seq approach [43].

Reasons for the absence of differential gene expression between ME/CFS patients and controls include (1) the lack of objective diagnostic testing for ME/CFS and reliance on subjective definitions, resulting in heterogeneity in the ME/CFS study cohort, (2) the lack of an immunological signature in ME/CFS that is detectable by transcriptomics, (3) localization of ME/CFS pathogenicity to a specific tissue (e.g. skeletal muscle or brain tissue) rather than blood, (4) heterogeneity of whole blood components with respect to the transcriptional signature, (5) multiple mechanisms underlying ME/CFS in different patients, complicating the identification of a unique signature. Dynamic shifts in viral abundance have been associated with immune cell dysregulation in setting of immunosuppression and obesity [12,13]; here, we observed no differences in viral abundance in ME/CFS patients following exercise.. In addition, detected viral transcripts were to DNA viruses commonly associated with chronic infection, including herpesviruses and anelloviruses. Detection of herpesviruses may suggest latent low-level infection of white blood cells, inflammatory reactivation, and/or active replication [44], while anelloviruses are considered non-pathogenic viral flora and have not yet been linked to any human disease [45]. Regardless, there were no differences in abundance of these viruses between ME/CFS and controls, and pre/post-exercise.

A limitation of the current study is the small size of the study cohort. However, a power analysis study reviewing 6 RNA-seq datasets measuring differential gene expression in human and mouse showed a study power >0.9 when comparing more than 10 samples per condition at a sequencing depth of 10–25 million reads per sample, 8–36% transcriptome mapping rate and comparing 5 differential expression calculation methods [46]. Our present longitudinal study design resulted in a higher transcriptome resolution thanks to increased transcriptome mapping (average, 78%), and increased differential expression accuracy by employing a multi-model approach correcting for small sample sizes. We anticipate our study to be adequately powered when comparing all CFS patients to all healthy controls, or all CFS samples pre- vs post-CPET. Nonetheless, given the lack of accurate and objective diagnostic markers for ME/CFS, if differences in gene expression are indeed present in one or more subsets of these patients, very large cohort studies may be required to identify these genes.

In conclusion, we observed no important differences in gene expression in ME/CFS patients over time and relative to controls in association with experimentally induced PEM. Previously reported differences in immunologic and metabolic function were derived from cross-sectional studies for which causation may not be inferred. Further progress in understanding ME/CFS may be dependent on nested analyses within prospective cohort studies and larger multi-center randomized clinical trials.

## Supporting information

S1 AppendixContains Figures A-F. **(Fig A)** Next-generation sequencing total read counts (grey bar), percentage of reads uniquely mapping to the human transcriptome (black diamond), and transcriptome coverage as the percentage of genes detected (red diamond). Two outlier samples with transcriptome coverage <40% were removed from subsequent human transcriptome analysis. **(Fig B)** Principal component analysis of the global gene expression shows no batch effect among the different sets of whole blood samples processed by RNA-seq analysis. **(Fig C).** Expression level dot plot and box plot of 6 differentially expressed genes (RPS12, SNORA27, RPL23A, HOXA9, NRON, LOC101192767) found when comparing CFS patients to controls at all time points. **(Fig D).** Expression level dot plot of LINC01158 found when comparing CFS patients to controls at day 7 only. **(Fig E).** Expression level dot plots of 6 differentially expressed genes found at day 1 (LOC105372441), day 3 (LOC100133050, PMS2P2), and day 7 (TMEM262, PRRP21, USP50) when comparing a subset of CFS patients with reduced peak ${\dot{\text{V}}\text{O}}_{2}$ at day 2 to CFS and controls with regular peak ${\dot{\text{V}}\text{O}}_{2}$. (**Fig F)**. Expression level dot plots of 9 differentially expressed genes found at day 2 (MBIP, SNORA32), day 3 (LOC100133050, PMS2P2, RNASE8), and day 7 (TMEM262, LINC1068, CREB3L1, HNF4A-AS1) when comparing a subset of CFS patients with test-retest effect compared to CFS and controls without test-retest effect.(DOCX)
