# Supplementary material for: Whole blood human transcriptome and virome analysis of ME/CFS patients experiencing post-exertional malaise following cardiopulmonary exercise testing
Source: PLoS One. 2019 Mar 21;14(3):e0212193. doi: 10.1371/journal.pone.0212193 (PMC6428308; doi:10.1371/journal.pone.0212193)
Supplement: S1 Appendix — Contains Figures A-F. (Fig A) Next-generation sequencing total read counts (grey bar), percentage of reads uniquely mapping to the human transcriptome (black diamond), and transcriptome coverage as the percentage of genes detected (red diamond). Two outlier samples with transcriptome coverage <40% were removed from subsequent human transcriptome analysis. (Fig B) Principal component analysis of the global gene expression shows no batch effect among the different sets of whole blood samples processed by RNA-seq analysis. (Fig C). Expression level dot plot and box plot of 6 differentially expressed genes (RPS12, SNORA27, RPL23A, HOXA9, NRON, LOC101192767) found when comparing CFS patients to controls at all time points. (Fig D). Expression level dot plot of LINC01158 found when comparing CFS patients to controls at day 7 only. (Fig E). Expression level dot plots of 6 differentially expressed genes found at day 1 (LOC105372441), day 3 (LOC100133050, PMS2P2), and day 7 (TMEM262, PRRP21, USP50) when comparing a subset of CFS patients with reduced peak V˙O2 at day 2 to CFS and controls with regular peak V˙O2. (Fig F). Expression level dot plots of 9 differentially expressed genes found at day 2 (MBIP, SNORA32), day 3 (LOC100133050, PMS2P2, RNASE8), and day 7 (TMEM262, LINC1068, CREB3L1, HNF4A-AS1) when comparing a subset of CFS patients with test-retest effect compared to CFS and controls without test-retest effect. (DOCX) [file pone.0212193.s001.docx]

**SUPPORTING INFORMATION**

**Whole blood human transcriptome and virome analysis of ME/CFS patients experiencing post-exertional malaise following cardiopulmonary exercise testing**

Jerome Bouquet^1^, Tony Li^1^, Jennifer L. Gardy^2,3^, Xiaoying Kang^3^, Staci Stevens^4^, Jared Stevens^4^, Mark VanNess^4^, Christopher Snell^4^, James Potts^5^, Ruth R. Miller^3^, Muhammad Morshed^6,7^, Mark McCabe^2^, Shoshana Parker^8^, Miguel Uyaguari^6^, Patrick Tang^9^, Ted Steiner^10^, Wee-Shian Chan^10^, Astrid-Marie De Souza^11^, Andre Mattman^7,12^, David M. Patrick^2,3^#, and Charles Y. Chiu^1,13^# for the Complex Chronic Disease Study Group

^1^Department of Laboratory Medicine, University of California San Francisco, CA, USA

^2^Communicable Disease Prevention and Control Services, British Columbia Centre for Disease Control (BCCDC), Vancouver, Canada

^3^School of Population and Public Health, University of British Columbia, Vancouver, Canada

^4^Workwell Foundation, Ripon, CA, USA

^5^Department of Pediatrics, Division of Cardiology, University of British Columbia, Vancouver, Canada

^6^British Columbia Centre for Disease Control Public Health Laboratory, Vancouver, Canada

^7^Department of Pathology and Laboratory Medicine, University of British Columbia, Vancouver, Canada

^8^Centre for Health Evaluation Outcome Sciences, Vancouver, Canada

^9^Department of Pathology, Sidra Medical and Research Center, Doha, Qatar

^10^Department of Medicine, Division of Infectious Diseases, University of British Columbia, Vancouver, Canada

^11^Division of Cardiology, British Columbia’s Children’s Hospital, Vancouver, Canada

^12^Adult Metabolic Disease Clinic, Vancouver General Hospital, Vancouver, Canada

^13^Department of Medicine, Division of Infectious Diseases, University of California, San Francisco, CA, USA

#co-corresponding authors
